# Fast Economic Development Accelerates Biological Invasions in China

**DOI:** 10.1371/journal.pone.0001208

**Published:** 2007-11-21

**Authors:** Wen Lin, Guofa Zhou, Xinyue Cheng, Rumei Xu

**Affiliations:** 1 Ministry of Education Key Laboratory for Biodiversity Sciences and Ecological Engineering, Beijing Normal University, Beijing, China; 2 College of Health Sciences, University of California at Irvine, Irvine, California, United States of America; University of Pretoria, South Africa

## Abstract

Increasing levels of global trade and intercontinental travel have been cited as the major causes of biological invasion. However, indirect factors such as economic development that affect the intensity of invasion have not been quantitatively explored. Herein, using principal factor analysis, we investigated the relationship between biological invasion and economic development together with climatic information for China from the 1970s to present. We demonstrate that the increase in biological invasion is coincident with the rapid economic development that has occurred in China over the past three decades. The results indicate that the geographic prevalence of invasive species varies substantially on the provincial scale, but can be surprisingly well predicted using the combination of economic development (R^2^ = 0.378) and climatic factors (R^2^ = 0.347). Economic factors are proven to be at least equal to if not more determinant of the occurrence of invasive species than climatic factors. International travel and trade are shown to have played a less significant role in accounting for the intensity of biological invasion in China. Our results demonstrate that more attention should be paid to economic factors to improve the understanding, prediction and management of biological invasions.

## Introduction

Biological invasion has recently become a serious ecological and economical problem in China where it is estimated that the 11 most serious invasive species have caused a loss of 57.4 billion Chinese Yuan (equivalent to ca.6.9 billion US dollars in 2002) per year to the Chinese economy [1]. It is commonly agreed that international transportation and trade have intensified the influx of invasive species (introduced accidentally or deliberately) by land, air, and sea from places that were formerly isolated [2]. Increased international exchange is accompanied by increased economic development and globalization; this is especially true in China where economic growth is unique in the last 3 decades.

Though some studies have discussed the impact of international trade and traffic, land use, and construction on the spread and occurrence of invasive species [3]–[12], so far there has been no quantified, statistical evidence on the relationship between economic development and biological invasion. Upon reviewing recent publications on biological invasions, we found that almost all papers concerning the mechanisms for the occurrence and spread of invasive species were related to biological factors; only a small proportion were related to the impact of climatic factors and even fewer addressed economic factors. Some discussions focused on the impact of biological invasions on economic loss, but not vice versa.

While biological factors determine the invasiveness of the alien species and the resistance of the invaded ecosystem, climatic factors determine the occurrence potential of the alien species in the new area. Aside from biological and climatic factors, economic factors have both direct and indirect impacts on biological invasions. Economic and other human factors influence the transportation and redistribution of invasive species populations; they are also responsible for disturbances of natural habitats that allow invasive species to establish. This topic has been overlooked, but needs to be heavily stressed and investigated. Our study examines the impact of economic factors on biological invasions combined with climatic factors to determine the relative importance of the two sets of factors.

## Results

**Figure 1:**
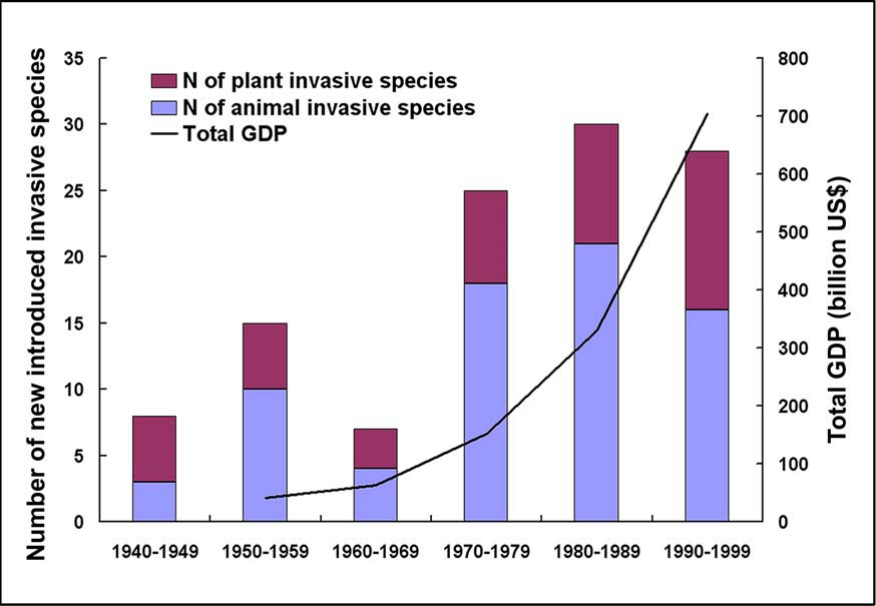
The total GDP and number of introduced invasive species into China. The total GDP is from 1959 to 1999, and the number of introduced invasive species into China is from 1940 to 1999.

Based on our survey of invasive species in China and economic data collected from the Chinese National Statistic Year Book [13], we have found that the rapid increase in the number per decade of newly introduced invasive species in China since the 1970s coincides with the sharp economic growth (as represented by Gross Domestic Production, GDP) experienced during the same period (Figure 1). Distributions of invasive plants and animals in each province are highly correlated (R = 0.815, F_1, 28_ = 55.823, P<0.001); therefore, we used invasive species to represent the combination of invasive plants and animals in the analysis outlined below.

**Figure 2:**
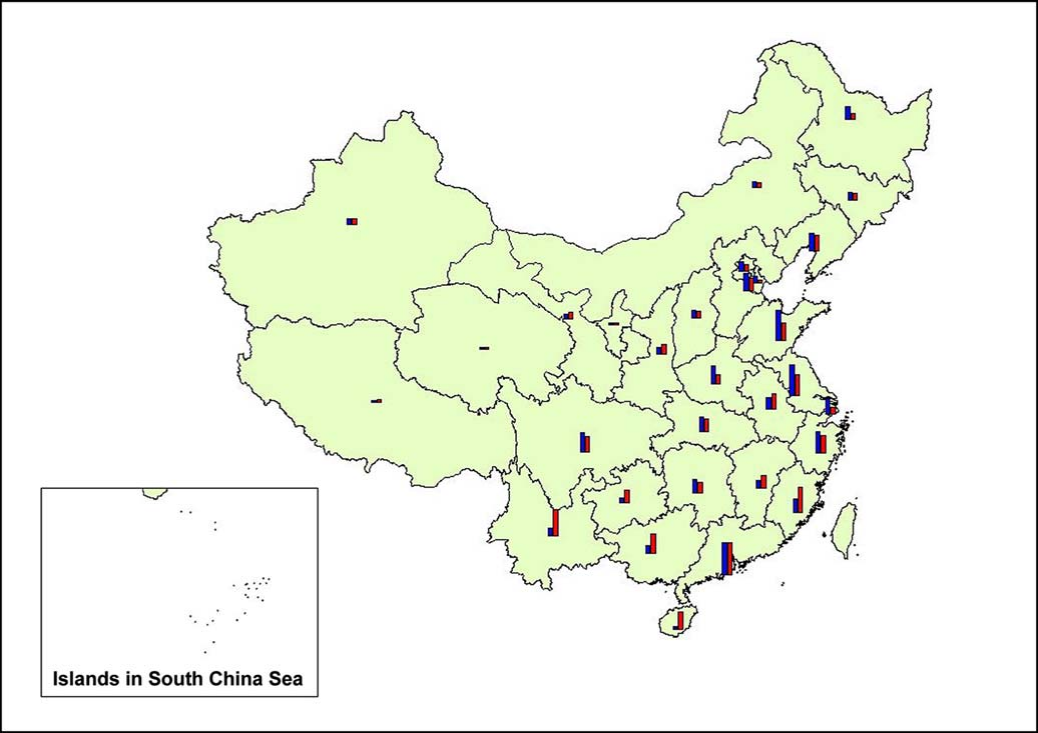
Distribution of the number of invasive species and average GDP in each province in China. The two bars (red bar for the number of invasive species, blue bar for average GDP from 1985 to 2004) are standardized with same height in Guangdong province which has the highest average GDP (76.7 billion USD) and the highest abundance of invasive species (117 species).

Spatial distribution of abundances of invasive species in each province (Figure 2) indicates a significant variation among provinces, with the more economically developed provinces in southern China and the coastal areas of eastern China having higher abundances of invasive species than provinces in inland and western China. For example, the number of invasive species in the pioneer southern opening province Guangdong, which is neighbouring Hong Kong and Macao was highest in China (117 species), whereas, the inland province Henan had only 38 invasive species. In comparison, the human population in Henan is about 1.6 times larger than that of Guangdong but produces only 53% of Guangdong's total GDP.

Through principal factor analysis (PFA), three principal components were selected; the first component has a contribution rate of 54.6 % of the total variance, the second component 19.5 %, and the third 13.4 % (Table 1). The first component consists mainly of economic variables in which residential construction and GDP have the highest load (0.938 and 0.933, respectively) as well as human population, the presence of primary, secondary and tertiary industries, freight and passenger traffic, investment in capital construction, and length of transportation routes. The second component includes mean annual temperature, mean January temperature and annual precipitation. The third principal component reflects the number of international tourists (as measured by foreign exchange earnings) and material flow (total import/export value of commodities).

**Table 1:** Result of the principal factor analysis of economic and climatic factors

Variables †		Factor loadings ‡		
		1	2	3
Floor Space of Buildings Through Capital Construction under Construction		0.938	0.085	0.122
Gross domestic product		0.933	0.089	0.254
Primary industry		0.918	0.161	−0.243
Freight traffic		0.907	−0.102	0.091
Secondary industry		0.899	0.037	0.314
Total population		0.897	0.123	−0.363
Tertiary industry		0.893	0.120	0.383
Investment in capital construction		0.889	0.083	0.395
Passenger traffic		0.879	0.220	−0.168
Length of transportation routes		0.749	0.079	−0.412
Mean annual temperature		0.287	0.941	0.063
Mean January temperature		0.174	0.929	0.021
Annual precipitation		0.282	0.872	0.109
Mean July temperature		0.444	0.625	0.109
Foreign exchange earnings		0.348	0.200	0.827
Imports and exports value of commodities		0.529	0.192	0.747
Rotated sums of squared loadings §	Eigenvalues	8.742	3.121	2.147
	% of variance	54.634	19.507	13.418
	Cumulative %	54.634	74.141	87.559

† Refer to Appendix S3 for details and units.

‡ Extraction method was principal component analysis.

§ Rotation method was Quartimax with Kaizer Normalization.

A multiple regression model was established between the number of invasive species and the factor scores of each province with a stepwise selection method. The first two principal components were selected by the stepwise method and they accounted for 72.5% of the total variance in number of invasive species, indicating a significant association between biological invasion and those factors (F_2, 27_ = 35.54, P<0.001). Economic factors proved most important, influencing the spatial variation and abundance of invasive species because they account for the highest value of variance (R^2^ = 0.378) (Table 2). This is especially true in northern China, where the abundance of invasive species was positively related with GDPs (Figure 2). Climatic conditions constitute the next most important component (R^2^ = 0.347) in influencing biological invasions. Higher temperatures and abundant rainfall in southern China may contribute to the great abundance of invasions in this area (Figure 2). International tourists and trade constitute the third group of principal factors. Because this third group of factors proved less important, it was eliminated during the stepwise selection process.

**Table 2:** Stepwise regression between number of invasive species and factor scores of the principal components

Variable entered by stepwise order	Regression		Analysis of variance (ANOVA)		
	Coefficients	R^2 ^ †	d.f.	F	Significance
Constant	3.729				
Factor score 1 ‡	0.352	0.378	1, 28	17.009	<0.0001
Factor score 2 ‡	0.337	0.725	2, 27	35.536	<0.0001

† Step by step cumulative R^2^.

‡ Factor Score 1 and Factor Score 2 correspond to principal components 1 and 2 in table 1.

## Discussion

From these results, we can summarize that aside from the basic biological factors which are the fundamental mechanisms for biological invasions, both economic developments and climatic variations are critical forces that govern the distribution and intensity of biological invasions in China. Climatic factors are ecologically important as they influence both habitat quality and population dynamics, and have been used as primary driving variables by almost all simulation models for biological invasions and risk analyses [14]. The argument presented here is that although economic factors are of high importance, they have been consistently overlooked in both research and in applications. The GDP in China increased from 896.4 billion Chinese Yuan in 1985 to 13687.6 billion in 2004 (current exchange). Investment in capital construction increased from 107.4 billion Yuan in 1985 to 2290.9 billion in 2003. With this huge economic growth and increase in investments, all aspects of construction (e.g., residential construction, transportation facilities, etc.) have exploded, exerting a significant impact on biological invasion.

### (1) Integrated biological, economical and climatic factors determine the occurrence and spread of invasive species

**Figure 3:**
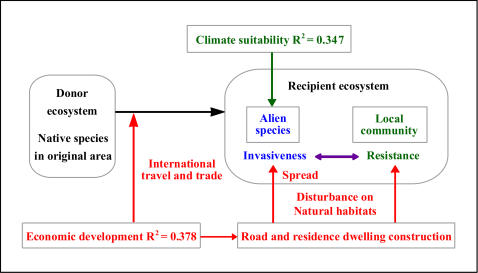
Relationship between biological, economical and climatic factors that determined the occurrence and spread of invasive species.

Biological invasion is a battle between the ability of the alien species to dominate its new environment and the resistance of the local community (Figure 3). Climatic conditions determine the potential geographic range where the alien species could establish its populations. Increase in economic development enhances international trade and travel that transport alien species to new areas. Economic development also brings about road and building constructions that in turn modifies natural habitats, enhances the spread of invasive species, intensifies the loss of resistance from the local communities to the invasions, thus accelerating biological invasions. Biology, meteorology and economy are the three legs of the tripod that constitutes the basis for understanding and predicting biological invasions.

If the assumption is true that economic development is highly correlated with scientific activity, then biological invasions might be more likely be reported in scientific literature in times and places where economic development is high which may influence the strength of the relationship discussed in this paper. Therefore, we have also analyzed two economic factors reflecting the strength of scientific activities: total funds and total expenditures in the field of natural sciences and technology. But both of these factors do not have significant linear correlation with invasive species (p = 0.877 and 0.896, respectively). The development of science, as another aspect of economic development, does not have much impact on the spread and redistribution of invasive species.

### (2) Holistic multidisciplinary systematic approaches are urgently required to understand the mechanisms of successful biological invasions

Enserink [15] stated: “As a tidal wave of exotic species transforms environments worldwide, ecologists are scrambling to predict where and when new invaders may strike”. This paper indicated that economic factors may help to partially predict where new invaders may strike.

In the academic field of invasion biology, much discussion and debate has concentrated on the mechanisms of successful biological invasions. One course concentrated on the life history traits of the invasive species [16]–[20]; another on the vulnerability of local communities to invasive species. These aspects include many hypotheses such as those of enemy release [2], [21]–[23], diversity resistance [2], [24], [25], evolution of improved competitive ability [26], niche opportunity [27], environmental heterogeneity [28], and so forth. Hybridization between species or between disparate source populations may also serve as a stimulus for the evolution of invasiveness [29].

Although these theoretical approaches are essential for understanding the biological processes that determine the success of biological invasions, we should not neglect economic and anthropogenic factors in our quest to understand and predict biological invasions. Biological invasion is a complicated chain of processes, consisting of introduction, establishment, spread, and outbreak, in which each link is not just a natural process, but also a result of human activities [30]. The high complexity of the biological processes involved, compounded by the extreme stochasticity of human activities, makes the understanding and prediction of biological invasions a very difficult task [31]. We demonstrate with this research that both biological and economic factors need to be carefully investigated to “begin the essential task of transforming the study of invasions from a diffuse anecdotal subject to a predictive science” [32].

Biological invasion is a chain of processes where only success in every link leads to the final success of the invader. The invader must have a suitable vector to transport it to its new habitat, and there must be sufficient introductions to guarantee the frequency and amount of imported individuals. After arriving in the new habitat, the invader's aggressiveness must be strong enough to overcome the resistance of the recipient ecosystem's natural enemies and other competitors; there must be a sufficient amount of host plants if the invader is an herbivore; the local climate must be suitable; and there must be a sufficient amount of local traffic to assist the spread of the invader. In summary, it is the integrated effect of numerous biological, climatic and economical factors that determine the success of the invasion. Thus, reconsidering the various hypotheses, a question arises: can one hypothesis fully explain the success of a biological invasion? For example, if in the new area, there are no specific natural enemies acting on the invader, it may partly explain why the invader can establish its population successfully. However, this view addresses only one of the links in the chain of processes. To explore the mechanisms for explaining successful invasions, other than analytical approaches, holistic multidisciplinary systematic approaches are directly needed.

### (3) Increasing GDP requires being “greener” and the problem of invasive species requires economic solutions

Economic development accelerates biological invasion, more invasions cause more ecological and economic damage. Obviously, we cannot stop invasions by slowing economic growth. Fast economic growth is welcomed and desirable to meet social requirements and to improve the quality of life of growing human populations. “The causes of the problem of invasive alien species are primarily economic and, as such, require economic solutions.” [6], what we need is a “greener” GDP that incorporates the goal of minimizing biological invasions. With the results obtained in this paper, we can conclude the following:

(a) Our data analysis demonstrated that there is a significant linearly relationship (p = 0.002) between residential construction and the number of invasive species. In the principal components analysis (PCA), it has the largest load within the first component (0.938). Indeed, we did not expect that the parameter of construction would play such an important role. However, it is not hard to explain. In the past 10 years, residential construction in china increased at the average rate of 15.3% per year. It is reported that nearly half of the world's buildings under construction are located in China [33]. Such rapid increase in residential buildings and expansion of small towns reduced the amount of existing habitats and also increased their fragmentation. At the end of 2020, construction area in China is predicted to increase by one fold, which is 68,600 million square meters [33]. It is not difficult to surmise that the rapid development of construction will have a significant impact on biological invasions in future decades. What's more, this growth will become one of the largest trials to the progress of China's urbanization. Therefore, it makes sense that we should implement ecological city planning now, and at the same time, prevent future habitat dislocation from deforestation due to increased construction.

(b) Other than primary industry, which has direct correlation with biological factors, secondary and tertiary industries also show significant correlation with the number of invasive species (p = 0.001, 0.000, respectively). Within the first component, they have the load of 0.899 and 0.893 respectively. Industrialization is necessary for the development and modernization of China, and China has made many achievements in the progress of industrialization. However, industrialization has also brought about numerous problems in the areas of ecology, energy and environment. At present, the fast development of Chinese industry is at the expense of large amounts of resources and rapid deterioration of its environment. In 2003, the overall output of industrial lumber was 47.59 million m^3^ compared with 12.33 million m^3^ in 1952, an increase of 4 folds. Such an enormous output can not only seriously destroy existing habitats, but also greatly increase the risk of diffusion of invasive species during transportation. In order to draw a reasonable balance among economic development, the human population, natural resources and the environment, we should implement sustainable industry now to protect and preserve our ecological systems.

(c) Within the first component, the load of freight traffic ranked fourth, while the load of passenger traffic was slightly less. Both of them has significant linearly correlation with invasive species (p = 0.004 and 0.001, respectively). Freight traffic and passenger traffic mainly occurred between provinces. Increased travel by domestic passengers and freight traffic over these 20 years further distributed invasive species across the country. Augmenting inter-province inspection and quarantine should be further stressed for restricting the spread of invasive species.

Foreign exchange earnings, imports and exports value of commodities are effective parameters of the third component. Their load to the third factor is 0.827 and 0.747 respectively. Both of them has significant linearly relationship with the number of invasive species (p = 0.020 and 0.001 respectively). Globalization of commerce increased the possibility of long-distance transport of invasive species all over the world. The introduction of many invasive species can be tracked with the routes of certain imported goods, such as wooden packing materials and live nursery stock, etc. [30], [34] Enhancement of supervision on economy and commerce related invasive pathways is the most effective way to reduce invasion frequency and diffusion rates, especially regarding important ports of import and export. The likelihood that a community will have received immigrant species is greatly due to its proximity to a seaport or other major point of entry and also the frequency, speed, and mode of dispersal of the immigrants themselves [35]. Our data showed that there are 24 immigrant species first discovered in Hong Kong that were then spread to the mainland. Intensifying inspection at some important areas of import and export, such as Shanghai, Hong Kong, Beijing and Guangdong will prove a cost-efficient way to reduce the incident of invasive species in China.

More research is clearly needed to clarify the major economic causes of biological invasions, which would result in more specific measures being taken, allowing China to strive for a greener GDP without slowing the course of economic developments. However, because of our current lack of understanding on how to achieve this ecology-economy balance, the solution is still largely unknown.

### (4) The potential value of expanding this research to other scales

Herein, we have provided a case study for investigating the impact of international trade and traffic, construction and climatic factors on the spread and distribution of invasive species both quantitatively and statistically. This study can be expected to expand on both larger and smaller scales. On the worldwide scale, we can investigate the similarities and differences of the major human factors which affect the distribution of invasive species in various countries or regions, especially those with different levels of economic development, to find common patterns and rules. Combined with the data from international trade and traffic, this will further clarify our understanding of the spread of invasive species. For instance, in a country with a low rate of economic development, the economy mainly relies on export of raw materials and typically imports fewer commodities, so the spread and distribution of invasive species there will likely be different from what we might find in more developed countries. On the other hand, on a smaller scale such as that of provinces, cities, or counties, this investigation may help local governments to establish effective measures for monitoring, prevention and control of invasive species.

## Materials and Methods

### Invasive species data collection

We constructed a list on known invasive species in China, based on published articles and books (Appendix S1 and Appendix S2). An alien species is defined as an invasive species only if it satisfies the following conditions: (1) has a known origin, (2) the time and location of its introduction into China or the first report of its occurrence there is known, and (3) it has caused environmental and/or economic damage in China. At the country level, the decadal scale total number of invasive species refers to the number of species causing reported damage in a specific year regardless of the number of localities reporting damage, i.e., multi-locality damage was counted as one. We investigated the number of invasive species in each of the 30 provinces (including autonomous regions and municipalities) in mainland China, where standardized and compatible data exists. Number of invasive species at the provincial level was the number of invasive species that has been reported to occur in that specific province since 1980, regardless of incidents of damage or outbreaks and the exact year of outbreak, i.e., multi-year damage was treated as one.

### Economic and climatic data collection

We collected economic and climatic data from 1985 to 2004 from the Chinese National Statistic Yearbook [13], published from 1986 to 2005. We converted the two different currencies (Yuan and USD) into USD by using the yearly average exchange rate of RMB Yuan against USD Dollars. Based on linear and log-linear regressions between economic variables and the number of invasive species in each province, 23 variables to represent different aspects of economic growth and development, and 4 variables to represent climatic variability in each province in mainland China were selected (Appendix S3). The mean values of these variables were used for data analysis.

### Data analysis

Principal factor analysis was carried out on these economic and climatic variables. The number of principal components we selected is based on Kaiser criteria. After analysis using Quartimax with Kaisier normalization rotation, we further removed those variables with absolute load <0.5. The remaining variables were subject to final principal factor analysis and a factor score for each province was given accordingly. A multiple regression model was established between the number of invasive species and the factor scores of each province, through stepwise selection method with p = 0.10 entering and p = 0.05 removing criteria.

## Supporting Information

Appendix S1 (0.16 MB DOC)

Appendix S2 (0.18 MB DOC)

Appendix S3 (0.04 MB DOC)
