# Supplementary material for: Fast Economic Development Accelerates Biological Invasions in China
Source: PLoS One. 2007 Nov 21;2(11):e1208. doi: 10.1371/journal.pone.0001208 (PMC2065902; doi:10.1371/journal.pone.0001208)
Supplement: Appendix S1 — (0.16 MB DOC) [file pone.0001208.s001.doc]

**Appendix S1. List of Invasive animal species**

| No. | Scientific name |
| --- | --- |
| 1 | *Acanthoscelides obtectus* (Say) |
| 2 | *Acanthoscelides pallidipennis* (Motschulsky) |
| 3 | *Achatina fulica* (Bowditch) |
| 4 | *Agrilus mali* Matsumura |
| 5 | *Anoplolepis gracilipes* (Smith) |
| 6 | *Anthrenus picturatus* Solskij |
| 7 | *Anthrenus verbaci* (Linnaeus) |
| 8 | *Aphanostigma piri* (Cholodkorsky) |
| 9 | *Aphelenchoides ritzemabosi* (Schwartz) Steiner & Buhrer |
| 10 | *Apis mellifera ligustica* Spinola |
| 11 | *Araecerus fasciculatus* (DeGeer) |
| 12 | *Asphondylia ervi* Rübsaamen |
| 13 | *Axionicis insignis* Pascoe |
| 14 | *Bactrocera cucurbitae* (Coquillett) |
| 15 | *Balanus amphitrite* (Darwin) |
| 16 | *Balanus eburneus* Gould |
| 17 | *Balanus improvisus* (Darwin) |
| 18 | *Bemisia tabaci* (Gennadius) |
| 19 | *Blattella germanica* (Linnaeus) |
| 20 | *Branta canadensis* (Linnaeus) |
| 21 | *Brontispa longissima* (Gestro) |
| 22 | *Bruchophagus gibbus* (Boheman) |
| 23 | *Bruchus pisorum* (Linnaeus) |
| 24 | *Bruchus rufimanus* Boheman |
| 25 | *Bufo marinus* (Linnaeus) |
| 26 | *Bugula neritina* (Linnaeus) |
| 27 | *Bugula stolonifera* Ryland |
| 28 | *Bursaphelenchus xylophilus* (Steiner & Buhrer) Nickle |
| 29 | *Cacatura sulphurea* (Gmelin) |
| 30 | *Callopora lineata* (Linnaeus) |
| 31 | *Callosciurus erythraeus thai* (Kloss) |
| 32 | *Callosobruchus analis* (Fabricius) |
| 33 | *Callosobruchus maculatus* (Fabricius) |
| 34 | *Callosobruchus phaseoli* (Gyllenhal) |
| 35 | *Carposina niponensis* (Walsingham) |
| 36 | *Celleporella hyalina* (Linnaeus) |
| 37 | *Cherax quadricarinatus* (Von Martens) |
| 38 | *Ciona intestinalis* (Linnaeus) |
| 39 | *Clarias* *gariepinus* (Burchell) |
| 40 | *Coleophora laricella* (Hübner) |
| 41 | *Contarinia sorghicola* (Coquillet) |
| 42 | *Cosmopolites sordidus (*Germar) |
| 43 | *Crepidula onyx* Sowerby |
| 44 | *Crisia eburneo-denticulata* Smitt |
| 45 | *Cryptotermes domesticus* (Haviland) |
| 46 | *Cryptotermes dudleyi* (Bank) |
| 47 | *Cryptotermes havilandi* (Sjostedt) |
| 48 | *Cydia inopinata* (Heinrich) |
| 49 | *Cydia pomonella* (Linnaeus) |
| 50 | *Cylas formicarius* (Fabricius) |
| 51 | *Dendroctonus valens* LeConte |
| 52 | *Ditylenchus* *dipsaci* (Kuhn) Filipjev |
| 53 | *Dysmicoccus brevipes* (Cockerell) |
| 54 | *Ephestia kuehniella* (Zeller) |
| 55 | *Eriosoma lanigerum* (Hausmann) |
| 56 | *Frankliniella occidentalis* (Pergande) |
| 57 | *Gambusia affinis* (Baird & Girard) |
| 58 | *Hemiberlesia pitysophila* Takagi |
| 59 | *Heterobostrychus aequalis* (Waterhouse) |
| 60 | *Hydroides elegans* (Haswell) |
| 61 | *Hyphantria cunea* (Drury) |
| 62 | *Hypostomus punctatus* Valenciennes |
| 63 | *Icerya aegyptiaca* (Douglas) |
| 64 | *Icerya purchasi (*Maskell) |
| 65 | *Ictalurus punctatus* (Rafinesque) |
| 66 | *Ictiobus cyprinellus* (Valenciennes) |
| 67 | *Incisitermes minor* (Hagen) |
| 68 | *Labeo rohita* (Hamilton) |
| 69 | *Lehmannia valentiana* (Férussac) |
| 70 | *Lichenopora radiata* (Audouin & Savigny) |
| 71 | *Liriomyza huidobrensis* (Blanchard) |
| 72 | *Liriomyza sativae* Blanchard |
| 73 | *Liriomyza trifolii* (Burgess) |
| 74 | *Lissorhoptrus oryzophilus* Kuschel |
| 75 | *Lithobates catesbeiana* (Shaw) |
| 76 | *Lithobates grylio* (Stejneger) |
| 77 | *Lithobates heckscheri* (Wright) |
| 78 | *Matsucoccus matsumurae* (Kuwana) |
| 79 | *Mayetiola destructor* (Say) |
| 80 | *Membranipora savartii* (Audouin) |
| 81 | *Meromyza saltatrix* (Linnaeus) |
| 82 | *Microporella orientalis* Harmer |
| 83 | *Micropterus salmoides* (Lacepède) |
| 84 | *Molgula manhattensis* (Dekay) |
| 85 | *Mus musculus* Linnaeus |
| 86 | *Myocastor coypus* (Molina) |
| 87 | *Mytilopsis sallei* (Recluz) |
| 88 | *Nematus melanaspis* Hartig |
| 89 | *Neocerambyx raddei* (Biessig et Solsky) |
| 90 | *Octodonta nipae* (Maulik) |
| 91 | *Oncorhynchus mykiss* (Walbaum) |
| 92 | *Ondatra zibethicus* (Linnaeus) |
| 93 | *Opogona sacchari* (Bojer) |
| 94 | *Oracella acuta* (Lobdell) |
| 95 | *Oreochromis aureus* (Steindachner) |
| 96 | *Oreochromis mossambicus* (Peters) |
| 97 | *Oreochromis niloticus niloticus* (Linnaeus) |
| 98 | *Pectinophora gossypiella* (Saunders) |
| 99 | *Periplaneta americana* (Linnaeus) |
| 100 | *Periplaneta australasiae* (Fabricius) |
| 101 | *Pheidole megacephala* (Fabricius) |
| 102 | *Phthorimaea operculella* (Zeller) |
| 103 | *Piaractus brachypomum* (Cuvier) |
| 104 | *Pomacea canaliculata* (Lamarck) |
| 105 | *Procambarus clarkii* (Girard) |
| 106 | *Quadrastichus erythrinae* Kim |
| 107 | *Rattus norvegicus* (Berkenhaut) |
| 108 | *Rattus rattus* (Linnaeus) |
| 109 | *Rattus tanezumi* Temminck |
| 110 | *Reticulitermes speratus* (Kolbe) |
| 111 | *Rhabdoscelus lineaticollis* (Heller) |
| 112 | *Rhynchophorus ferruginus* (Oliver) |
| 113 | *Rhyzopertha dominica* (Fabricius) |
| 114 | *Schizoporella unicornis (*Johnston*)* |
| 115 | *Sciaenops ocellatus* (Linnaeus) |
| 116 | *Sciurus vulgaris exalbidus* Pallas |
| 117 | *Sitophilus granarius* (Linnaeus) |
| 118 | *Sitophilus oryzae* (Linnaeus) |
| 119 | *Solenopsis invicta* Buren |
| 120 | *Sphaeroma walkeri* Stebbing |
| 121 | *Sternochetus olivieri* (Faust) |
| 122 | *Strongylocentrotus intermedius* (Agassiz) |
| 123 | *Styela canopus* (Savigny) |
| 124 | *Tetranychus urticae* Koch |
| 125 | *Trachemys scripta elegans* (Wied-Neuwied) |
| 126 | *Trialeurodes vaporariorum* (Westwood) |
| 127 | *Tribolium castaneum (*Herbst*)* |
| 128 | *Tricellaria occidentalis* (Trask) |
| 129 | *Trichoglossus haematodus* (Linnaeus) |
| 130 | *Trogoderma granarium* Everts |
| 131 | *Tubulipora flabellaris* Fabricius |
| 132 | *Viteus vitifoliae* (Fitch) |
| 133 | *Watersipora subtorquata* (d'Orbigny) |
| 134 | *Zabrotes subfasciatus* (Boheman) |
| 135 | *Zoobotryon verticillatum* (Delle Chiaje) |
