# Supplementary material for: Fast Economic Development Accelerates Biological Invasions in China
Source: PLoS One. 2007 Nov 21;2(11):e1208. doi: 10.1371/journal.pone.0001208 (PMC2065902; doi:10.1371/journal.pone.0001208)
Supplement: Appendix S2 — (0.18 MB DOC) [file pone.0001208.s002.doc]

**Appendix S2. List of Invasive plant species (introduced into China after 1900)**

| No | Scientific Name |
| --- | --- |
| 1 | *Abutilon crispum* (L.) Medicus |
| 2 | *Acanthospermum australe* (L.) Kuntze |
| 3 | *Adhatoda vasica* Nees. |
| 4 | *Aegilops squarrosa* L. (=*A. tauschii* Coss.) |
| 5 | *Ageratum houstonianum* Miller |
| 6 | *Alexandrum catenella* Balech |
| 7 | *Alopecurus aequalis* Sobol. |
| 8 | *Alternanthera paronychioides* St. Hil. |
| 9 | *Alternanthera philoxeroides* (Mart.) Griseb |
| 10 | *Alternanthera pungens* H. B. K. |
| 11 | *Amaranthus albus* L. |
| 12 | *Amaranthus caudatus* L. |
| 13 | *Amaranthus hybridus* L. |
| 14 | *Amaranthus lividus* L. |
| 15 | *Amaranthus polygonoides* L. |
| 16 | *Ambrosia artemisiifolia* L. |
| 17 | *Ambrosia trifida* L. |
| 18 | *Anredera cordifolia* (Tenore) Steenis |
| 19 | *Aster subulatus* Michx. |
| 20 | *Axonopus compressus* (Swartz) Beauv. |
| 21 | *Bidens alba* (L.) DC. |
| 22 | *Bidens frondosa* L. |
| 23 | *Brachiaria eruciformis* (J. E. Smith) Griseb |
| 24 | *Brachiaria mutica* (Forsk.) Stapf |
| 25 | *Bromus unioloides* H.B.K. |
| 26 | *Bryophyllum pinnatum* (L. f.) Oken |
| 27 | *Cabomba caroliniana* A. Gray |
| 28 | *Calonyction aculeatum* (L.) House |
| 29 | *Cassia alata* (L.) Roxb. |
| 30 | *Cassia mimosoides* L. |
| 31 | *Cassia occidentalis* L. |
| 32 | *Cassia tora* L. |
| 33 | *Celosia argentea* L. |
| 34 | *Cenchrus echinatus* L. |
| 35 | *Cenchrus incertus* M. A. Curtis |
| 36 | *Centaurea diffusa* Lam. |
| 37 | *Chaetoceros concavicornis* Mangin |
| 38 | *Coronopus didymus* (L.) J.E. Smith |
| 39 | *Cosmos sulphureus* Cav. |
| 40 | *Crassocephalum crepidioides* (Benth.) S. Moore |
| 41 | *Cucumis melo* L. var. *agrestis* Naud. |
| 42 | *Cuphea balsamona* Cham. & Schltdl. |
| 43 | *Cyclindrotheca closterium* Reimann & Lewin |
| 44 | *Cyclospermum leptophyllum* (Pers.) Sprague ex Britton & P. Wilson |
| 45 | *Desmodium tortuosum* (Sw.) DC. |
| 46 | *Duranta repens* L. |
| 47 | *Ehrharta erecta* Lam. |
| 48 | *Eichhornia crassipes* (Mart.) Solms |
| 49 | *Eleusine indica* (L.) Gaertn. |
| 50 | *Erechtites hieracifolia* (L.) Raf. ex DC |
| 51 | *Erechtites valerianaefolia* (Wolf ex Rchb.) DC |
| 52 | *Erigeron philadelphicus* L. |
| 53 | *Eupatorium adenophorum* Spreng |
| 54 | *Eupatorium cannabinum* L. |
| 55 | *Eupatorium odoratum* L. |
| 56 | *Euphorbia dentata* Michx. |
| 57 | *Euphorbia maculata* L. |
| 58 | *Euphorbia nutans* (Lag.) Small |
| 59 | *Flaveria bidentis* (L.) Kuntze |
| 60 | *Galinsoga parviflora* Cav. |
| 61 | *Galinsoga quadriradiata* Ruiz et Pavon |
| 62 | *Gaura parviflora* Lehm. |
| 63 | *Geranium carolinianum* L. |
| 64 | *Gomphrena celosioides* Mart. |
| 65 | *Heliotropium europaeum* Ait. |
| 66 | *Hordeum jubatum* L. |
| 67 | *Hyptis brevipes* Poir. |
| 68 | *Hyptis rhomboidea* M. Martens & Galeotti |
| 69 | *Indigofera suffruticosa* Mill. |
| 70 | *Ipomoea cairica* (L.) Sweet. |
| 71 | *Ipomoea triloba* L. |
| 72 | *Jatropha curcas* L. |
| 73 | *Lepidium campestre* (L.) R. Br. F. glabratum (Lej. Et Court.) Thell. |
| 74 | *Lepidium densiflorum* Schrad. |
| 75 | *Lepidium virginicum* L. |
| 76 | *Lolium multiflorum* Lam. |
| 77 | *Lolium temulentum* L. |
| 78 | *Lolium temulentum* L. *var longiaristatum* Parnell |
| 79 | *Lolium temulentum* L.*var. arvense* Bab. |
| 80 | *Macroptilium atropurpureum* (DC.) Urb. |
| 81 | *Megathyrsus maximus* (Jacq.) B. K. Simon & S. W. L. Jacobs |
| 82 | *Melosira cancellate* |
| 83 | *Mikania micrantha* H.B.K. |
| 84 | *Mimosa invisa* Mart. |
| 85 | *Mimosa sepiaria* Benth. |
| 86 | *Nicandra physalodes* (L.) Gaertn. |
| 87 | *Nitzschia delicatissima* Cleve. |
| 88 | *Oenothera drummondii* Hook. |
| 89 | *Oenothera erythrosepala* Borb. |
| 90 | *Oenothera laciniata* Hill. |
| 91 | *Oenothera rosea* L'Hér. ex Aiton |
| 92 | *Parthenium hysterophorus* L. |
| 93 | *Paspalum conjugatum* P. J. Bergius |
| 94 | *Paspalum dilatatum* Poir. |
| 95 | *Paspalum fimbriatum* H.B.K. |
| 96 | *Pennisetum purpureum* Schumach. |
| 97 | *Pennisetum setosum* (Swartz) Rich. |
| 98 | *Peperomia pellucida* (L.) Kunth |
| 99 | *Peridinium perardiforme* |
| 100 | *Perocentrum balticum* (Lohmann) Loeblich |
| 101 | *Phalaris minor* Retz. |
| 102 | *Phalaris paradoxa* L. |
| 103 | *Pharbitis nil* (L.) Choisy |
| 104 | *Phleum pratense* L. |
| 105 | *Physalis peruviana* L. |
| 106 | *Physalis pubescens* L. |
| 107 | *Phytolacca americana* L. |
| 108 | *Pilea microphylla* (L.) Liebm. |
| 109 | *Pinnularia viridis* Bohm |
| 110 | *Plantago aristata* Michx. |
| 111 | *Plantago virginica* L. |
| 112 | *Poa compressa* L. |
| 113 | *Praxelis clematidea* R. M. King & H. Rob. |
| 114 | *Prorocentrum minimum* (Pavillard) Schiller |
| 115 | *Prorocentrum sigmoides* Bohm |
| 116 | *Pseudelephantopus spicatus* (J. ex Aub.) Gleason |
| 117 | *Pyrethrum parthenifolium* Willd. |
| 118 | *Ranunculus arvensis* L. |
| 119 | *Reseda lutea* L. |
| 120 | *Rhynchelytrum repens* (Willd.) C.E.Hubbard |
| 121 | *Richardia brasiliensis* Gomes |
| 122 | *Robinia pseudoacacia* L. |
| 123 | *Saponaria officinalis* L. |
| 124 | *Sclerocarpus africanus* Jacq. ex Murray |
| 125 | *Scrippsiella trochoidea* (Stein) Loeblich III |
| 126 | *Sechium edule* (Jacq.) Swartz |
| 127 | *Sesbania cannabina* (Retz.) Pers. |
| 128 | *Sicyos angulatus* L. |
| 129 | *Silene latifolia* Poir. ssp. alba (Mill.) Greuter & Burdet |
| 130 | *Silene vulgaris* (Moench) Garcke |
| 131 | *Silphium perfoliatum* L. |
| 132 | *Silybum marianum* Graetn. |
| 133 | *Solanum rostratum* Dunal. |
| 134 | *Solanum sarachoides* Sendtn. |
| 135 | *Solanum surattense* Burm. f. |
| 136 | *Solidago canadensis* L. |
| 137 | *Soliva anthemifolia* (Juss.) R. Br. |
| 138 | *Sonneratia apetala* Buch.-Ham. |
| 139 | *Sorghum halepense* (L.) Pers. |
| 140 | *Sorghum sudanenses* (Piper) Stapf |
| 141 | *Spartina alterniflora* Loisel |
| 142 | *Spartina anglica* C.E. Hubb. |
| 143 | *Spermacoce latifolia* Aubl. |
| 144 | *Stachytarpheta jamaicensis* (L.) Vahl |
| 145 | *Stellaria apetala* Ucria |
| 146 | *Symphytum officinale* L. |
| 147 | *Synedrella nodiflora* (L.) Gaertn. |
| 148 | *Tagetes minima* L. |
| 149 | *Tephrosia candida* (Roxb.) DC. |
| 150 | *Tithonia diversifolia* A. Gray |
| 151 | *Tridax procumbens* L. |
| 152 | *Triodanis biflora* (Ruiz & Pavón) Greene |
| 153 | *Triodanis perfoliata* (L.) Nieuwl. |
| 154 | *Veronica arvensis* L. |
| 155 | *Veronica hederaefolia* L. |
| 156 | *Veronica persica* M. Pop. |
| 157 | *Vetiveria zizaniodes* (L.) Nash |
| 158 | *Wedelia trilobata* Klitchc. |
| 159 | *Xanthium glabrum* (DC.) Britton. |
| 160 | *Xanthium italicum* Moretti |
| 161 | *Xanthium spinosum* L. |
| 162 | *Zinnia peruviana* (L.) L. |
