# Supplementary material for: Fast Economic Development Accelerates Biological Invasions in China
Source: PLoS One. 2007 Nov 21;2(11):e1208. doi: 10.1371/journal.pone.0001208 (PMC2065902; doi:10.1371/journal.pone.0001208)
Supplement: Appendix S3 — (0.04 MB DOC) [file pone.0001208.s003.doc]

**Appendix S3: List of variables used for analysis (units)**

| Gross Domestic Product (100 million USD) |
| --- |
| Primary Industry (100 million USD) |
| agriculture (100 million USD) |
| forestry (100 million USD) |
| husbandry (100 million USD) |
| fishery (100 million USD) |
| Secondary Industry (100 million USD) |
| industry (100 million USD) |
| construction (100 million USD) |
| Tertiary Industry (100 million USD) |
| Investment in Capital Construction (100 million USD) |
| Floor Space of Buildings Through Capital Construction under Construction (10,000 square meters) |
| Freight Traffic (10,000 tons) |
| Imports Value of Commodities by the Places of Their Destination and Exports Value of Commodities by the Place of Their Origin in China (USD 1,000) |
| Imports (USD 1,000) |
| Foreign Exchange Earnings by Region (USD 10,000) |
| Passenger Traffic by Region (10,000 persons) |
| Length of Transportation Routes (km) |
| Railways (km) |
| Highways (km) |
| Total Funds of State-owned Research and Development Institutions and Information and Literature Institutions at and above County Level in the Field of Natural Sciences and Technology(USD 10,000) |
| Total Expenditures of State-owned Research and Development Institutions and Information and Literature Institutions at and above County Level in the Field of Natural Sciences and Technology(USD 10,000) |
| Population (10,000 persons) |
| Mean January temperature (^o^C) |
| Mean July temperature (^o^C) |
| Mean annual temperature (^o^C) |
| Annual precipitation (mm) |
